# Research on express service defect evaluation based on semantic network diagram and SERVQUAL model

**DOI:** 10.3389/fpubh.2022.1056575

**Published:** 2022-12-02

**Authors:** Suishan Gu, Kangyu Wang, Lianyue Gao, Jun Liu

**Affiliations:** ^1^School of Business and Management, Jilin University, Changchun, Jilin, China; ^2^The 716 Research Institute of China Shipbuilding Industry Group Co., Ltd., Lianyungang, Jiangsu, China

**Keywords:** express service defects, text mining, semantic network diagram, SERVQUAL model, LDA topic model

## Abstract

This paper constructs a defect evaluation model of express service, uses the text mining methods of web crawler, SVM (Support Vector Machine) emotion analysis and LDA (Linear Discriminant Analysis) topic model to capture and clean up the online negative comment data of express service, establishes a semantic network diagram, and uses LDA topic model to extract the characteristic words of defect topic. Based on SERVQUAL model, it can classify the subject characteristic words of express service defects from the dimensions of tangibility, reliability, responsiveness, assurance, empathy and economy, etc., calculate the degree value and attention value of express service defects, and establish IPA model for defect mapping and identify the improvement direction. The evaluation model constructed in this paper has reference value for evaluating the defects of service industry and improving service quality. It is found that the “responsiveness” defect is the primary improvement direction, and the reliability, assurance and economy are the secondary improvement defects. Among them, the “responsiveness” defect has five improvement detail defects. The evaluation model constructed in this paper has reference value for evaluating the defects of service industry and improving service quality.

## Introduction

In the context of e-commerce, express delivery has become an essential service. At the same time, the emergence of many express logistics enterprises makes enterprises begin to pay attention to the improvement of service quality (1). The level of express service has a significant impact on the satisfaction of e-commerce customers (2). However, at present, the relevant research on express service quality mostly focuses on the establishment of express service quality evaluation model and the main factors affecting express service quality (3–5), and there is little direct research on express service defects. Identifying and monitoring service defects is very important for enterprises to meet customer needs and improve service quality (6, 7). User generated content (UGC) has good autonomy, universality and interactivity, and can truly reflect user evaluation (8). Text mining technology can transform the text content in UGC into the latest and valuable information (9–11). Semantic network diagram is a tool for text mining, which can be applied to the qualitative evaluation of express service quality (12, 13). SERVQUAL model is a quantitative evaluation tool, which not only measures the quality of service provision, but also shows users' views on the services provided (14, 15). This paper uses text mining method to build an express service defect evaluation model based on semantic network diagram SERVQUAL model, which helps the express industry identify the key elements that need to be improved in order to improve the service quality.

## Literature review

### Defects of express service

Service defects arise when the service level provided by enterprises is lower than customers' expectations (16, 17). Frequent express service failures will not only lead to the business loss and reputation damage of express companies, but also cause customers' emotional anxiety and have a negative impact on their satisfaction, resulting in negative word-of-mouth and complaints. For example, the uneven distribution of resources in Postal Express outlets affects the distribution efficiency and causes timeliness defects (18); Express service often has defects of convenience and trust (19); Accuracy and timeliness greatly affect the quality of express service (5); Delivery time and accuracy are the biggest obstacles in the delivery chain (20). Diversified problems or complaints from customers include relatively high distribution price, unreasonable signing process, timeliness of distribution, delayed delivery, damage and loss of goods, signing before inspection, etc. (21, 22); Even staff professionalism and uniform dress (23).

Most of the above studies use second-hand data obtained by questionnaire to identify quality defects, and lack first-hand data support from the perspective of customer perception.

### Text mining and its application

Web text mining is the representation, feature extraction, content summary, classification, clustering, association analysis, semantic analysis and trend prediction of online text content (24). Text mining helps people make appropriate decisions by applying different artificial intelligence algorithms (25).

Using text mining technology, we can identify quality defects by obtaining the public feeling data of the network platform. For example, text mining technology can extract the basic mode of e-commerce logistics from a large number of unstructured documents and formulate the strategy of e-commerce logistics management (26). The poor efficiency of port logistics process can also be estimated by combining data-driven technology—process mining (PM), social network analysis (SNA) and text mining (27). Text mining technology is used to identify cross-border logistics service elements and customers' feelings (28), and LDA model can also be used to identify service attributes (29). When studying the logistics service of fresh e-commerce, scholars also use text mining model based on convolution neural network to analyze the logistics service elements related to customer satisfaction (30). In the identification of key problems of service quality, scholars try to use text mining technology to analyze the medium and poor evaluation data in the online customer review data of e-commerce platform, and explore the prominent problems in logistics (31); Or analyze the negative network reputation of household products and services with the help of text mining technology to identify the key factors leading to logistics service errors (32); Or use SVM method to classify and analyze the positive and negative emotions of customer comments of express service (33, 34).

When identifying defects, the above studies mostly define them by the frequency of problems mentioned in the evaluation data, without considering the classification requirements of service defect types, or often directly define the problems as service quality defects, lacking research depth.

This paper establishes an express service defect evaluation model based on semantic network diagram- SERVQUAL model, and provides a service defect identification method combined with text mining tools and service quality type definition. By capturing the first-hand data of customers' feelings on the public platform, we can identify service defects, and the identified service defects are closely combined with customers' objective feelings.

## Establishment of evaluation model of express service defects

### Evaluation framework of express service defects

**Figure 1:**
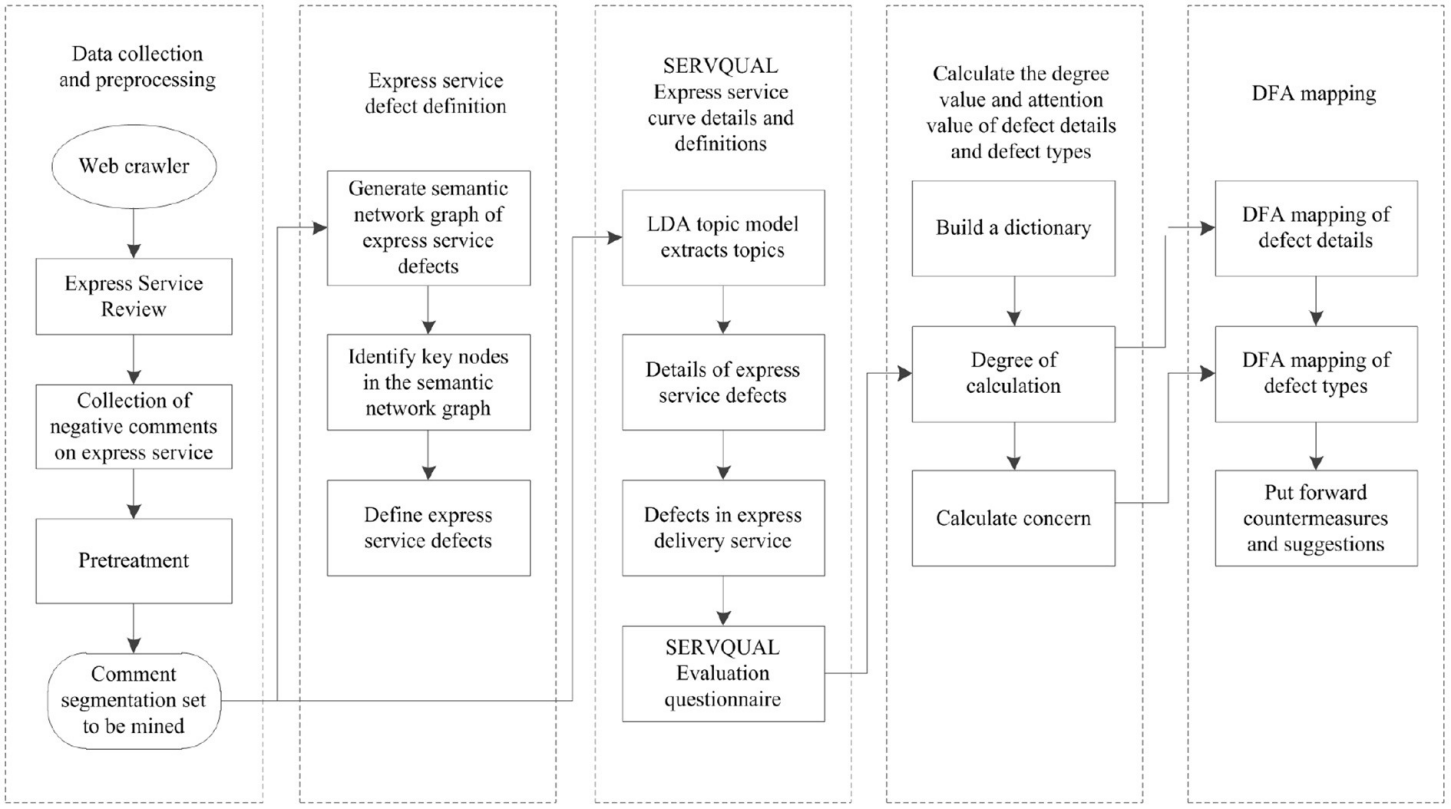
Evaluation framework of express service defect based on semantic network diagram-SERVQUAL model.

This paper constructs the defect evaluation model of express service based on semantic network diagram-SERVQUAL model (Figure 1). The key steps are as follows:

(1) Python is used to capture the express service comments from popular social platform and shopping platform which have sufficient and appropriate data resource, and SVM emotion analysis method is used to classify the express service comment set. It includes noun extraction, comment de duplication, mechanical compression and de duplication, deleting non text content, deleting short sentence comments, comment participles and removing stop words, extracting nouns and gerunds, and filtering irrelevant words to form a comment participle set to be mined.
(2) ROSTCM6 software is used to mine the comment word set, generate the semantic network diagram of express service defects, determine the key defect nodes, and define the express service defects.
(3) LDA topic model is applied to extract the topics of the comment word set to be mined, identify the express service problems, map them to the express service defect types in SERVQUAL model, and form a detailed list of service defects.
(4) The emotional dictionary and degree adverb dictionary of express service defects are constructed, to calculate the degree value and attention value of express service defects, and make z standardization to prepare for IPA mapping.
(5) Defect focus analysis (DFA) model is constructed according to the principle of IPA. The defect types of express service and relevant defect details of SERVQUAL model are mapped to DFA to identify key defects and defect details, so as to provide reference for improvement.

### Calculation of degree value and attention value of express service defects

#### Construction of emotion dictionary and degree adverb dictionary of express service defects

According to the negative emotion dictionary in HowNet and NTUSD and the derogatory dictionary (35–37) proposed by Li Jun, an emotion dictionary with express service defects is formed (Table 1). At the same time, referring to the degree adverb dictionary in HowNet, the common degree adverbs are divided into five categories, and the degree adverbs of each level are given decreasing weight value α respectively (Table 2).

**Table 1:** Emotion dictionary (part).

Emotion dictionary	Annoying, miserable, no one listens, disgusting, vomiting, unbearable, bad attitude, regret, ignore, shit, slow, mentally retarded, shutdown, dam it, too bad, fuck, garbage, fake, anxious, no, scolding, dying, despicable, unable to get through, die out, angry, failed to get through

**Table 2:** Dictionary of degree adverbs of express service defects (part).

**Grade**	**Items**	**Weight value (α)**
I. Extreme	Don't overdo, exceed, exceed heterodyne, get ahead, over, over	2.5
II. Very	However, many, invincible, heavy, strange, great, many, more, most, especially	2
III. More	No big deal, more, further, more, also, even, compare, further	1.5
IV. Some	Bit by bit, more or less, strange, also, more or less, some, slightly	1.0
V. Insufficiently	A little, not much, chatting, mild, weak, slightest, slight, tiny, relative	0.5

#### Calculation of service defect degree value

Firstly, taking a comment in the word segmentation set of express service defect comments as the analysis unit, the emotion value of this comment is obtained by matching the feature word, emotion dictionary and degree adverb dictionary; each comment in the defect comment segmentation set is analyzed, to get the emotional value of n comments; Finally, it can calculate the average emotion value of n comments to get the emotion value of feature words. The calculation formula is as follows:


(1) $$sen_{i}=E\left(\sum_{j=1}^{n}\alpha comment\_sen_{i,j}\right)\text{​},i=1,2,...,n;j=1,2,...,n$$

Where, *sen*_*i*_ represents the emotional value of a feature word; α represents the weight value of degree adverbs; If the comment contains characteristic words and emotional words, then *comment*_*sen*_*i,j*_ value is 1, otherwise it is 0.

In this paper, for each defect detail of express service, the average emotion value of the five feature words with the highest probability of occurrence is calculated as the defect degree value. The calculation formula is as follows:


(2) $$\begin{array}{l} Defect_{i}=\left(\overset{5}{\underset{j=1}{\sum }}sen_{i,j}\right),i=1,2,...,n;j=1,2,3,4,5 \end{array}$$

The calculation formula of defect degree value of defect type of express service is as follows:


(3) $$\begin{array}{l} D_{i}=E\left(\overset{5}{\underset{j=1}{\sum }}Defect_{i,j}\right),i=1,2,...,n;j=1,2,3,4,5 \end{array}$$

#### Calculation of attention value of defect index and dimension

The amount of comments on the topic indicates that the customer's attention to the topic is basically consistent with the result of customer perception (38), and the amount of reading on the topic also reflects the customer's attention to the topic (39). Therefore, this paper uses two kinds of indicators: comments and reading to calculate the attention value of express service defects.

Firstly, the feature word-comment participle word pair is matched. If the word pair is matched, the comment amount of the feature word is increased by 1; Secondly, it makes the feature word traverse each comment in the defect comment segmentation set. If the number of matched word pairs is x, the comment amount of the feature word is x. For the detailed defect, this paper calculates the sum of the comment amount of the five characteristic words as the comment amount of the defect, and the calculation formula is as follows:


(4) $$\begin{array}{l} C_{i}=\overset{5}{\underset{j=1}{\sum }}x_{i,j},i=1,2,...,n;j=1,2,3,4,5 \end{array}$$

Where, *C*_*i*_ represents the comment amount of a defect index of express service, and *x*_*i,j*_ represents the comment amount of a feature word under the defect.

Secondly, the rules for calculating the reading amount of characteristic words are the same as above, and the formula for calculating the reading amount of defect index of express service is as follows:


(5) $$\begin{array}{l} R_{i}=\overset{5}{\underset{j=1}{\sum }}M_{i,j},i=1,2,...,n;j=1,2,3,4,5 \end{array}$$

Where, *R*_*i*_ represents the reading amount of a defect index of express service, and *M*_*i,j*_ represents the reading amount of a feature word under the defect.

Finally, the comments and readings of the defect index of express service are given the weight value *β*_*i*1_, *β*_*i*2_, respectively, and the two are multiplied by the corresponding weight value and summed to obtain the attention value of the defect index of express service. The calculation formula is as follows:


(6) $$\begin{array}{l} Focus_{i}=\beta_{i1}\times C_{i}+\beta_{i2}\times R_{i} \end{array}$$

$$\begin{array}{l} Where,\beta_{i1}=\frac{C_{i}}{C_{i}+R_{i}},\beta_{i2}=\frac{R_{i}}{C_{i}+R_{i}}. \end{array}$$

The calculation formula for the attention value of the defect dimension of express service is as follows:


(7) $$\begin{array}{l} F_{i}=E\left(\overset{n}{\underset{j=1}{\sum }}Focus_{i,j}\right),i=1,2,...,n;j=1,2,...,n \end{array}$$

### IPA mapping between defect detail and defect type

**Figure 2:**
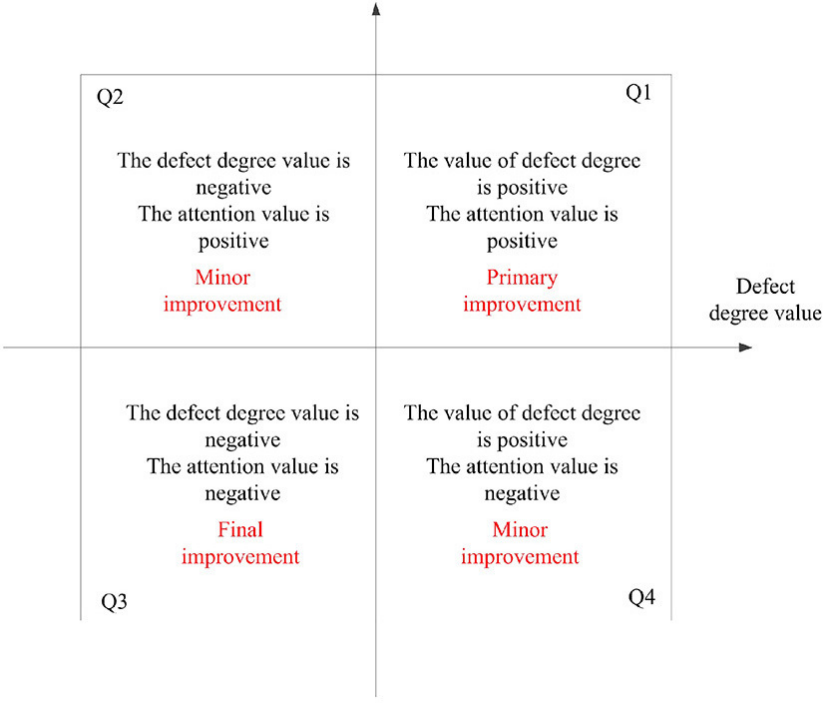
DFA model.

Importance performance analysis (IPA) model, is called importance- performance analysis model, divides products or services into four quadrants by evaluating the importance and performance of products or services, so as to formulate improvement strategies (40, 41). In this paper, the defect degree and attention degree are regarded as the two dimensions of IPA model, and the model is defined as defect focus analysis (DFA) model. The meaning represented by its four quadrants is shown in Figure 2. *Z* standardization of the results of defect degree value and attention value is conducive to dimensional elimination (42, 43). After mapping, key improvement defects can be directly identified to improve improvement efficiency.

## Application research

### Data collection and preprocessing

#### Collection of negative comment data of express service

This paper selects the top social media platforms and shopping websites in China, including Baidu Post Bar, Microblog, Zhihu, Douban and Taobao, as the data source, crawls the express service comment data from 2020 to 2022, and obtains 198,674 data in total.

SVM emotion analysis method is applied to classify the emotion of express service comments, and the mature practice is used for reference: the crawled comments are marked with emotion, and 8,000 negative comments and 8,000 other comments are obtained; Secondly, 16,000 comments are divided into training set and test set according to the ratio of 4:1; Then, the SVM module under Gensiem library is used to generate SVM classifier. The classifier is trained with training set and test set. After multiple training, the accuracy, recall and *F*1 values are 93.2, 92.8, and 88.38% respectively. The classification accuracy is high and the training is completed; Finally, the trained SVM classifier is applied to the express service comment set, and 157,536 negative comment sets of express service are obtained.

#### Preprocessing of negative comment set of express service

**Figure 3:**
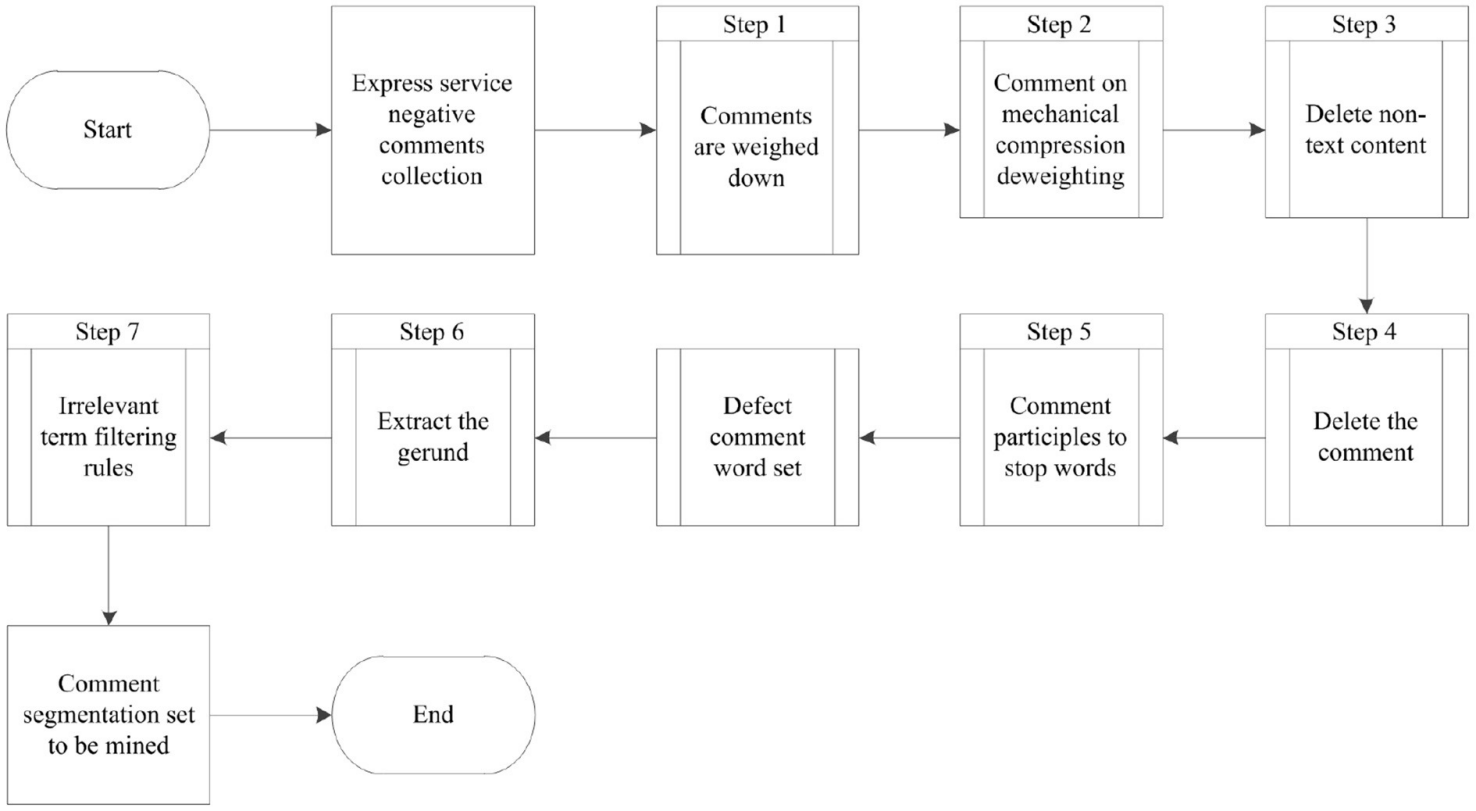
Processing process and result of negative comment set of express service.

The pre-process and results of negative comments on express service are shown in Figure 3. The whole processing includes weight reduction, mechanical compression weight removal, deletion of non text content, etc., and 133728 comments were obtained.

### Definition of express service defects

**Figure 4:**
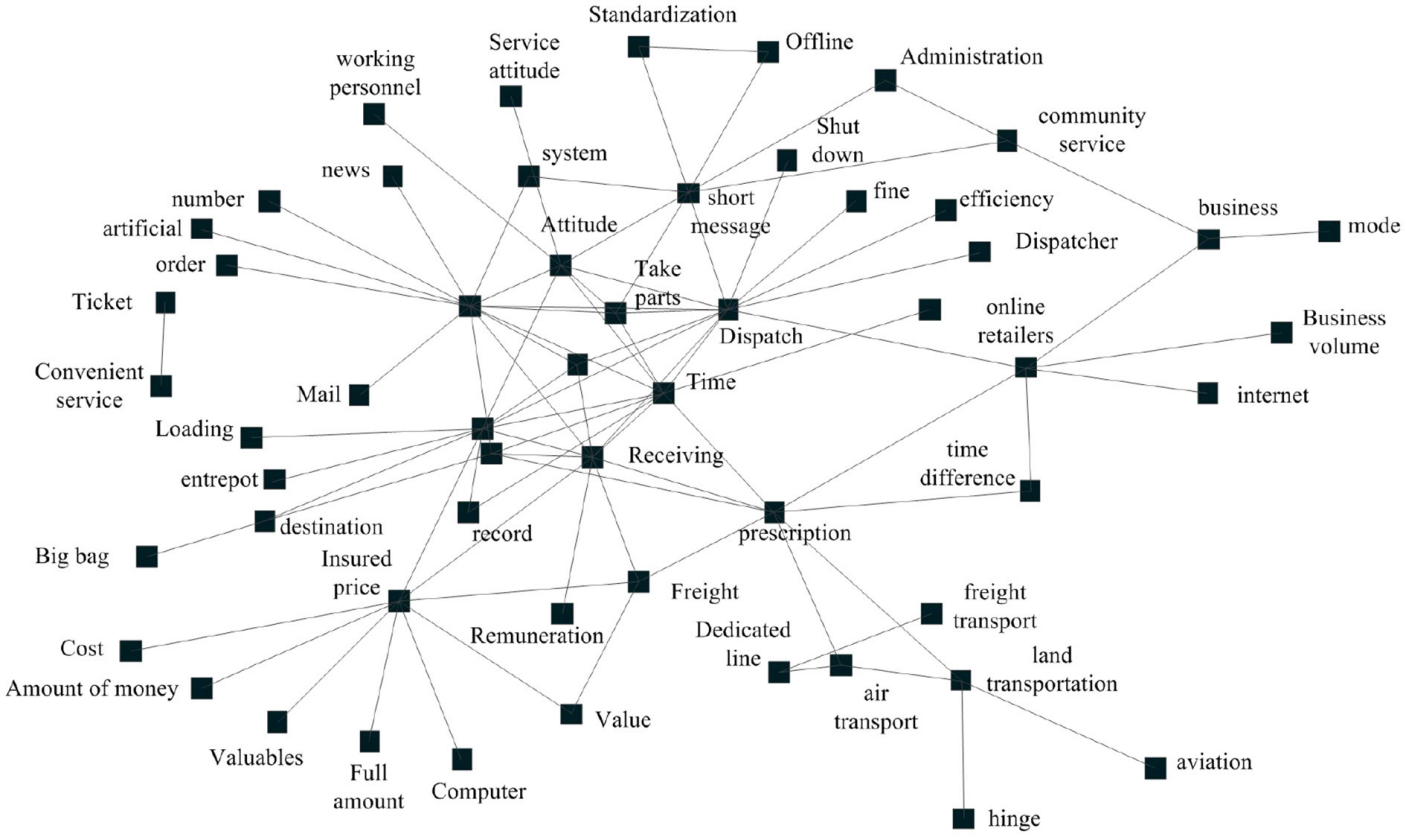
Semantic network diagram of defects in express service.

Using ROSTCM6 software, it can quickly analyze the comment word set to be mined, and generate the semantic network diagram of express service defects as shown in Figure 4. As can be seen from Figure 4, the number of arcs of time, information, dispatch, express, receipt and insurance is >9, and the number of arcs of timeliness, SMS, attitude, freight and pick-up is >3. They are all key defect nodes in the semantic network diagram, which are in an important position in the semantic network diagram of express service defects.

### Calculation of defect degree and attention of express service

#### Identification of defect details of express service items

**Figure 5:**
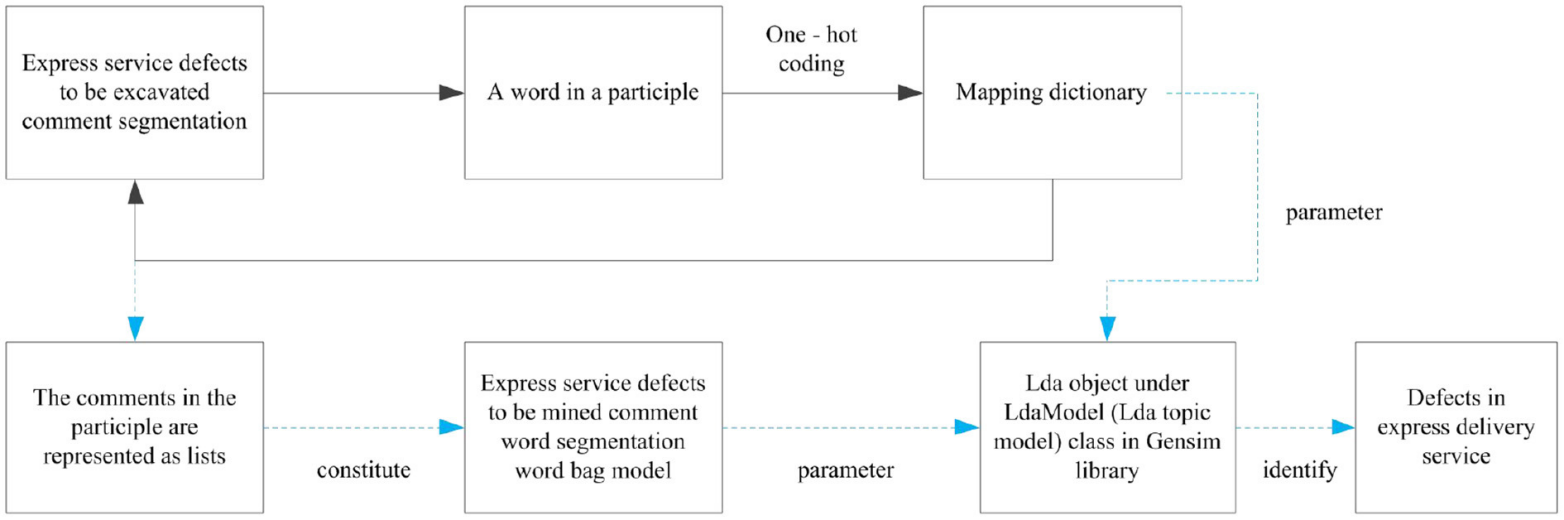
Process for identifying delivery service defects.

One hot coding is used to encode the word items in the comment word set of express service defects to be mined, generate a mapping dictionary, and then represent each comment as a list. The collection of all lists constitutes the word bag model of the comment word set of express service defects to be mined. Secondly, LdaModel (LDA topic model) under the framework of Gensim, a third-party extension package of python, is used to extract feature words from the word bag model. Five feature words with the highest probability under each topic are set to extract the topics of express service defects. The identified 22 express service defect topics are taken as the detail defects of express service in SERVQUAL evaluation model (Table 3). The extraction process is shown in Figure 5.

**Table 3:** 22 topics of defect of express service.

	**Defect of express service**	**Feature words**
1	Unreasonable charging price	Freight, express fee, public, pricing, opinions
2	Unsafe valuables	Insurance, valuables, computers, laptops, electronic products
3	Unreasonable compensation price	Compensation, compensation, suggestions, store bullying, price
4	Delayed receipt	Receiving, overdue, remuneration, reaction, offline
5	Reject large parts	Large goods, emotional, heavy goods, sent off, delivery
6	Untimely delivery by express	Express, delivery, express delivery, punctuality, attitude
7	Poor integrity of outer packaging	Violence, case, outer package, sorting, inspection
8	Failing to feed back customer's complaints in time	Labor, special lines, public complaints, comments, excuses
9	Failing to feed back the customer's requirements in time	Requirements, time limit, number, consultation, private letter
10	Poor quality of staff	Deception, mistakes, quality, service awareness, defects
11	Poor diversity of transportation modes	Aircraft, land transportation, air transportation, location, aviation
12	Slow transportation speed	Express, snail, express, efficiency, speed
13	Low intensity of preferential activities	Discount, method, message, strength, receipt
14	Poor corporate image	Staff, care, salary, relationship, treatment
15	Poor convenience of customer receiving	Receiving, reasonable, distance, route, walking
16	Delivering dangerous express	Contraband, send, sender, postal law, danger
17	Poor professional ability of staff	Staff, ability, business process, problem solving, specification
18	Poor quality of enterprise management	Responsibility, supervisor, operation, leadership, management
19	Small coverage of express outlets	Distribution center, destination, hub, scope, business point
20	Service facilities are not modernized	Facility, robot, logic, modernization, business department
21	Poor reliability of cold chain transportation	Cold chain, loading, absurdity, trust, loss
22	Information cannot be obtained in time	Information, system, SMS, record, background

#### Identify the defect type of express service

Learning from the SERVQUAL evaluation model built by Li and Hao (44) and Cao and Li (45), the detailed defects of express service are mapped to the corresponding service defect types, namely, tangibility, reliability, responsiveness, assurance, empathy and economy (Table 4).

**Table 4:** The results of the degree value and attention value of express service defect.

**Defect type**	**Degree detail**	**Degree value**	***Z*-value of degree**	**Attention value**	***Z*-value of attention**
A: Tangibility	I. Poor quality of staff	1.28	0.39	12,383	−0.26
	II. Poor diversity of transportation modes	1.15	−1.85	8,713	−0.72
	III. Poor quality of enterprise management	1.35	1.60	11,943	−0.32
	IV. Service facilities are not modernized	1.21	−0.81	11,036	−0.43
B: Reliability	I. Reject large parts	1.20	−0.99	13,453	−0.13
	II. Untimely delivery by express	1.30	0.74	38,963	3.07
	III. Poor integrity of outer packaging	1.29	0.56	20,138	0.71
	IV. Delivering dangerous express	1.22	−0.64	11,615	−0.36
C: Responsiveness	I. Delayed receipt	1.27	0.22	19,204	0.59
	II. Failing to feed back customer's complaints in time	1.30	0.74	11,498	−0.37
	III. Failing to feed back the customer's requirements in time	1.23	−0.47	12,524	−0.24
	IV Information cannot be obtained in time	1.38	2.12	34,518	2.51
D: Assurance	I. Unsafe valuables	1.31	0.91	20,601	0.77
	II. Slow transportation speed	1.22	−0.64	9,638	−0.60
	III. Poor professional ability of staff	1.28	0.39	13,276	−0.15
E: Empathy	I. Poor corporate image	1.24	−0.30	12,782	−0.21
	II. Poor convenience of customer receiving	1.24	−0.30	11,080	−0.42
	III. Small coverage of express outlets	1.16	−1.68	9,864	−0.58
	IV. Poor reliability of cold chain transportation	1.19	−1.16	13,920	−0.07
F: Economy	I. Unreasonable charging price	1.28	0.39	7,833	−0.83
	II. Unreasonable compensation price	1.33	1.25	5,142	−1.17
	III. Low intensity of preferential activities	1.23	−0.47	8,063	−0.80

#### Degree value and attention value of defect details and defect types

According to the calculation formula, the degree value and attention value of defect details and defect types of express service are calculated, respectively, and the values are *Z* standardized (Tables 4, 5).

**Table 5:** The results of degree value and attention value of express service defect.

**Defect dimension**	**Degree value**	***Z*-value of degree**	**Attention value**	***Z*-value of attention**
A: Tangibility	1.25	−0.35	11,019	−0.51
B: Reliability	1.25	−0.35	21,035	1.5
C: Responsiveness	1.30	1.41	19,436	1.18
D: Assurance	1.27	0.35	10,879	−0.53
E: Empathy	1.21	−1.77	11,911	−0.33
F: Economy	1.28	0.71	7,013	−1.31

#### DFA mapping of defect

**Figure 6:**
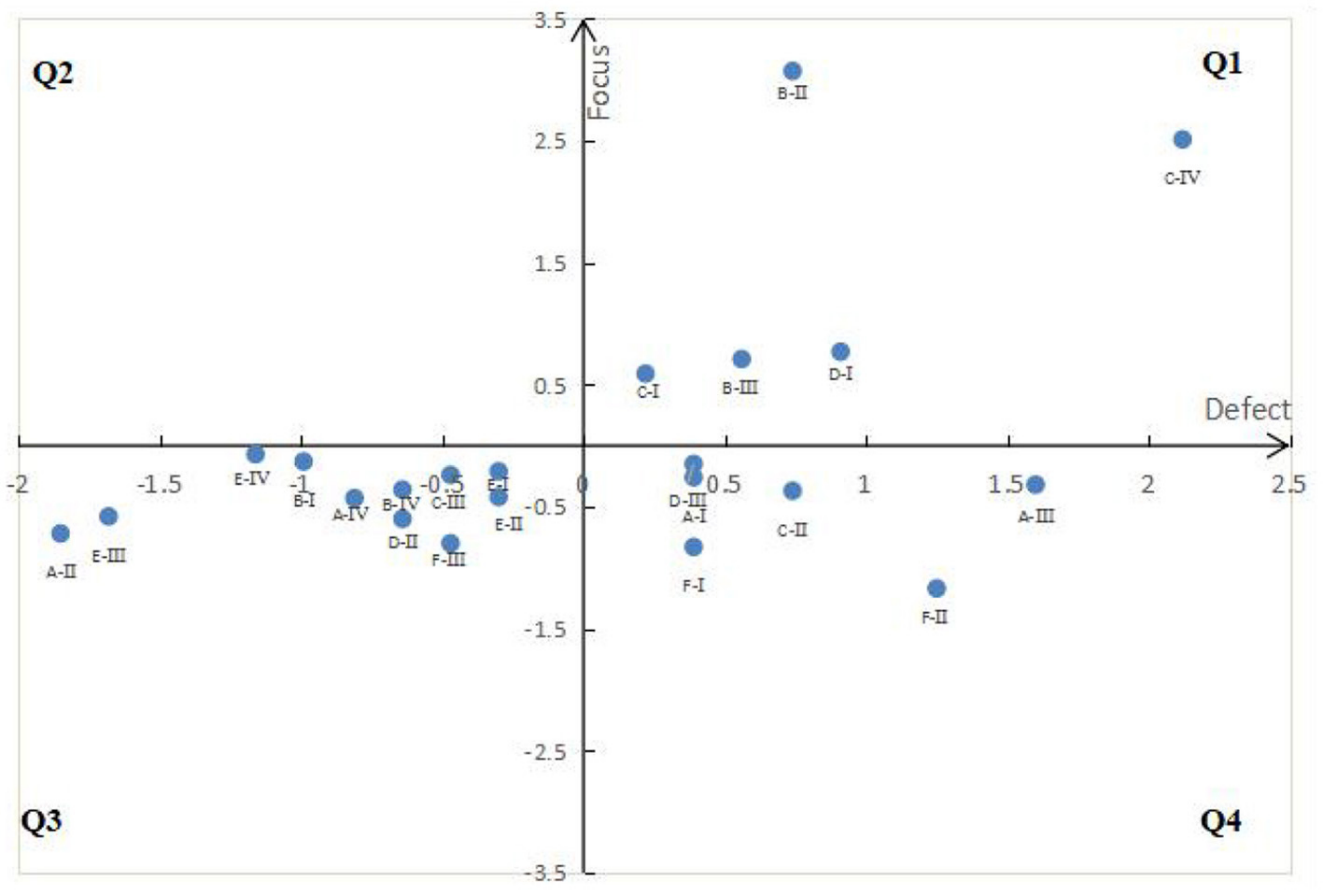
DFA diagram of defect detail.

**Figure 7:**
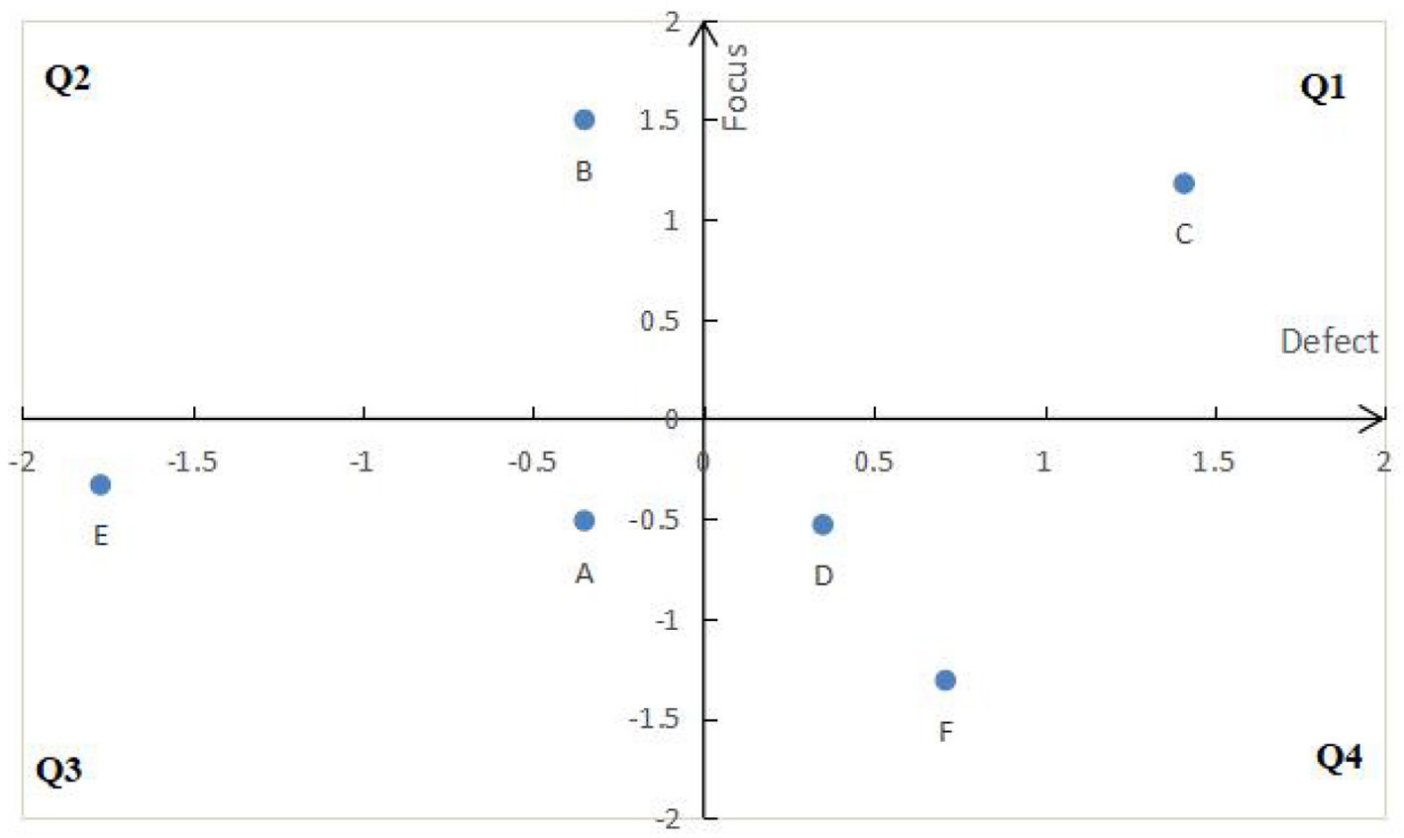
DFA diagram of defect type.

The defect degree value and attention value after z standardization are mapped to the quadrant diagram of DFA model, respectively (Figures 6, 7).

## Discussion

Through the evaluation model of express service defects based on semantic network diagram and SERVQUAL model, we have the following findings:

(1) Although defect details can been identified (as shown in Table 3) through text mining technology, but it is very efficient to classify defect details by using SERVQUAL model, and then can help the managers to formulate the service improvement policy.
(2) Using defect degree and defect concern degree, we can more effectively improve the express service quality to improve customer satisfaction.Those specific defects with low degree value and low attention value, such as A-I, A-III, C-II, D-III, F-I, F-II shown in Q4 of Figure 6, may not have priority to improve, because improving these defects don't meant quickly improving customer satisfaction. Similarly, D and F in in the Q4 of Figure 7.
(3) Combined with the quadrant positions of defect details and defect types in Figures 6, 7, the improvement order of service quality is determined, as shown in Table 6.

**Table 6:** Improvement order of defect dimension and defect index.

**Improvement order**	**Defect type**	**Defect detail**
Primary improvement	C: Responsiveness	B-II: The express delivery is not timely, B-III: Poor integrity of outer packaging, C-I: Untimely receipt, C-IV: Express information cannot be obtained in time, D-I: The valuables of the representative are not safe.
Secondary improvements	B: Reliability, D: Assurance, F: Economy	A-I: Poor quality of staff, A-III: Poor quality of enterprise management, C-II: Untimely customer feedback, D-III: Poor professional ability of staff, F-I: Unreasonable charge price, F-II: Unreasonable compensation price
Finally improvements	A: Tangibility, E: Empathy	11 (omitted)

According to the above table, the following improvement countermeasures are put forward:

(1) In terms of responsiveness, it should firstly improve the specific problems of untimely receipt and untimely access to express information, and then improve the complaint defects of untimely feedback to customers.
(2) In terms of assurance, it should firstly improve the unsafe defects of valuables, and then improve the poor professional ability of staff.
(3) In terms of economy, it is necessary to improve the unreasonable charge price and unreasonable compensation price.
(4) In terms of reliability, it is necessary to improve the delay of express delivery and the poor integrity of outer packaging.

## Conclusion

This paper constructs an evaluation model of express service defect based on semantic network diagram- SERVQUAL, analyzes the express comments on social platforms and shopping websites through text mining technology, extracts the theme by LDA model, comprehensively analyzes and quantitatively calculates the express service defects by using semantic network diagram and SERVQUAL model, identifies the main defects of express service, and puts forward the improvement direction. Through the data mining and calculation of China's express service, the effectiveness and practicability of this model are verified, which provides guidance for the express service industry to improve the service quality. The research of this paper can be applied to the defect identification of other service fields, such as aviation service, tourism service, catering service, medical service, insurance service, telecommunications service and so on.

The research of this paper has the following limitations: firstly, it takes the negative comments of online comments as the data source, but does not consider eliminating malicious comments, which may reduce the objectivity of the data source; Secondly, when measuring the attention value of express service defects, only two indicators of comment volume and reading volume are selected, without considering other factors; Thirdly, the effect of improvement countermeasures was not demonstrated to prove the accuracy of defect identification. Future research work can be deployed in the above aspects.

## Data availability statement

The original contributions presented in the study are included in the article/supplementary material, further inquiries can be directed to the corresponding author/s.

## Author contributions

SG and KW: writing—original draft preparation. LG and JL: editing data curation and supervision. All authors contributed to the article and approved the submitted version.

## Conflict of interest

Author JL was employed by the 716 Research Institute of China Shipbuilding Industry Group Co., Ltd. The remaining authors declare that the research was conducted in the absence of any commercial or financial relationships that could be construed as a potential conflict of interest.

## Publisher's note

All claims expressed in this article are solely those of the authors and do not necessarily represent those of their affiliated organizations, or those of the publisher, the editors and the reviewers. Any product that may be evaluated in this article, or claim that may be made by its manufacturer, is not guaranteed or endorsed by the publisher.
